# Art Therapy and Its Impact on Mood and Emotional States in Pediatric Hematology Oncology Units: Translation and Validation of the Italian Version of the Arts Observational Scale (ArtsObS)

**DOI:** 10.3390/healthcare13151851

**Published:** 2025-07-29

**Authors:** Marianna Avola, Enrica Garibaldi, Milena La Spina, Andrea Di Cataldo, Giovanna Russo, Luca Lo Nigro, Maria Montanaro, Dorella Scarponi, Angela Militello, Clara Raciti, Federica Maio, Antonella Agodi, Martina Barchitta, Paola Adamo, Soani Duca, Davide Massidda, Momcilo Jankovic, Giulia Zucchetti, Cinzia Favara Scacco

**Affiliations:** 1 LAD ETS Cure&Care in Pediatric Oncology (Non-Profit Organization—NPO), 95125 Catania, Italy; garibaldienrica@gmail.com (E.G.); angelamilitello76@gmail.com (A.M.); clara.raciti@hotmail.com (C.R.); federicamaio7@gmail.com (F.M.); cinzia.favara@ladonlus.org (C.F.S.); 2 Unit of Physical Medicine and Rehabilitation, Santa Marta and Santa Venera Hospital, 95024 Acireale, Italy; 3 Pediatric Hematology Oncology Unit, Department of Clinical and Experimental Medicine, University of Catania, 95123 Catania, Italy; mlaspina@unict.it (M.L.S.); adicata@unict.it (A.D.C.); diberuss@unict.it (G.R.); lucalonigro1968@gmail.com (L.L.N.); 4 Pediatric Hematology and Oncology Unit, Santissima Annunziata Hospital, 74121 Taranto, Italy; maria.montanaro@libero.it; 5 Pediatric Medicine Unit, IRCSS AOU, 40138 Bologna, Italy; dorella.scarponi@aosp.bo.it; 6 Department of Medical and Surgical Sciences and Advanced Technologies “GF Ingrassia”, University of Catania, 95125 Catania, Italy; antonella.agodi@unict.it (A.A.); mbarchitta@unict.it (M.B.); 7 Lene Thun Foundation ETS (Non-Profit Organization—NPO), 39100 Bolzano, Italy; paola.adamo@thun.it (P.A.); soaniduca@gmail.com (S.D.); 8 Kode S.R.L., 56122 Pisa, Italy; d.massidda@kode-solutions.net; 9 Maria Letizia Verga Foundation, IRCCS San Gerardo Hospital, 20900 Monza, Italy; momcilo@libero.it; 10 Pediatric Oncohematology, Regina Margherita Children’s Hospital, 10126 Turin, Italy; giulia.zucchetti@unito.it

**Keywords:** art therapy, pediatric, oncology, psychology, test, translation

## Abstract

**Background/Objectives**: Art therapy is a psychotherapeutic technique that involves the creation of tangible visual arts and represents a coping strategy to support children with cancer. Evaluating the effects of such activities on children with cancer is essential for providing evidence of the value that creativity holds within healthcare systems. A dedicated tool for assessing the creative process is the Arts Observational Scale (ArtsObS), focusing on mood and emotional states as key indicators of psychosocial well-being. This study aims to validate a translated version of the ArtsObS in the Italian language. **Methods**: The translation process followed recommendations for translation and cultural adaptation. The distribution properties of the scores, internal consistency, sensitivity to change, reliability, and convergent validity were assessed through observations conducted by two different evaluators. **Results**: The ArtsObS in its Italian adaptation is proven to be an adequate tool for capturing changes following an intervention, with good internal consistency and low sensitivity to differences between operators. The analysis supports the reliability of the ArtsObS across different observers. **Conclusions**: The Italian ArtsObS is a valid and reliable instrument for evaluating the impact of art therapy on pediatric patients’ mood and emotional states. It provides a standardized tool for clinical and research settings to assess creative interventions in pediatric oncology.

## 1. Introduction

Art therapy is increasingly used in pediatric oncology as a supportive intervention to facilitate emotional expression through the creation of visual artworks [1,2]. The creative process enables children to externalize distressing emotions in a safe and nonverbal way [3,4,5], offering psychological benefits such as emotional regulation, cognitive stimulation, and enhanced interactions with peers and caregivers [6,7]. Several studies have demonstrated its potential in improving the psycho-emotional well-being of hospitalized children [7,8,9,10,11,12].

Despite the growing implementation of art-based programs in healthcare settings, the evaluation of their emotional impact remains limited by the lack of standardized, validated observational tools [13]. While measures of the overall quality of life (QoL) are commonly used, they may not capture the specific, short-term emotional changes targeted by art interventions. Reliable, context-sensitive tools are therefore needed to assess mood and engagement directly following creative activities.

The Arts Observational Scale (ArtsObS), developed by Fancourt and Poon [13], was designed to meet this need. It is a concise, structured instrument allowing trained observers to assess visible signs of mood and engagement before and after participation in art sessions. The original English version demonstrated solid psychometric properties, including high inter-rater reliability, good internal consistency, and sensitivity to emotional changes, making it suitable for use in both clinical and research contexts.

To date, no Italian version of the ArtsObS has been developed. The aim of this study is to translate, culturally adapt, and validate the ArtsObS within Italian pediatric hematology–oncology settings.

We hypothesize the following:

- The Italian version of the scale will demonstrate psychometric properties comparable to those of the original English version;
- It will be sensitive in detecting mood score variations following participation in an art-based intervention.

## 2. Materials and Methods

### 2.1. Methods

The translation process of the Arts Observational Scale (ArtsObS) from English to Italian was conducted by means of a forward translation and cultural adaptation of the psychometric instruments, followed by a back-translation step. Specifically, the Italian version was translated back into English by an independent bilingual translator unfamiliar with the original version. The back-translated version was then compared with the original English scale to identify and resolve any semantic or conceptual discrepancies, ensuring equivalence between the two versions.

The evaluation of the Italian version was carried out through an observational multicenter study on the whole Italian territory, conducted in pediatric hematology–oncology units.

### 2.2. The ArtsObS

The ArtsObS is a psychometric instrument designed to measure changes resulting from art-based interventions conducted in hospitalization contexts. It provides scores for three key conditions: mood, relaxation, and distraction. “Mood” refers to a person’s general emotional state, which can change quickly in response to external factors. It is a subjective feeling that can be influenced by various factors, such as the context, and is not necessarily linked to intense emotions [14]. “Relaxation” is a state of inner calmness and tranquility that can have a positive impact on a patient’s health and well-being in the hospital [15]. “Distraction” is a technique used in healthcare to reduce patient anxiety and pain levels. It works by utilizing the finite cognitive attentional resources, allowing for a reduction in negative sensations [16].

The ArtsObS tool uses Eccleston’s model [17] to rate the levels of distraction, considering three possible reactions: processing only the negative, only the positive, or both simultaneously.

The mood score is dual in nature, as it is collected both before the start of the activity and at its conclusion. From these two values, a third measure—mood variation—can be derived, calculated as the difference between the post-intervention mood score and the pre-intervention mood score.

A fourth condition, “ward effect,” refers to the activity’s capability to distract the patient or caregiver in the hospital (in-ward) context.

This structure allows for a nuanced evaluation of the impact of artistic activities on emotional states, contributing to the broader assessment of therapeutic interventions in clinical settings.

### 2.3. Methods for Validation of the Arts Observational Scale (ArtsObS)

Observations were conducted both in person and online during art therapy workshops offered to children and adolescents with oncological conditions and other pathologies—such as eating disorders, autoimmune and hematological diseases, and neurodevelopmental disorders—across Italy. All observers involved in the study participated in a preliminary online training course, structured into three interactive sessions for a total of 6 h. The training covered the use of the ArtsObS tool, including the operational definitions of each item, scoring criteria, and example cases. This process was made to ensure consistency in the evaluation process and reduce inter-rater variability. The ArtsObS was completed by the evaluator through a purely observational approach, making the process entirely non-intrusive.

To assess the reliability of the measurements, for a subset of participants, a primary evaluator was accompanied by a second independent evaluator.

The statistical analyses aimed at examining the validity of these measures and evaluating the ArtsObS included the following:

(a) Distributional properties of the scores: Assessing how the scores are distributed across the sample to ensure appropriate variability and sensitivity.
(b) Consistency of measurements (internal consistency): Evaluating the degree of coherence among items to confirm the scale’s reliability.
(c) Reliability of the measurements: Analyzing the degree of agreement between independent observers assessing the same variable.
(d) Convergent validity with an external criterion: Comparing the ArtsObS results with the FACES Pain Rating Scale (18) to establish concurrent validity by calculating the correlation (Pearson’s correlation coefficient).
(e) Sensitivity to change: Determining the scale’s ability to detect variations over time as a result of the intervention.

Data analyses were conducted using the R software environment (version 4.3.3), complemented by the psych package (version 2.4.3), ensuring rigorous and reproducible statistical processing.

### 2.4. Characteristics of the Sample

The ArtsObS was administered to a group of 395 pediatric patients and 61 family members (primarily 39 mothers and 13 fathers) who took part in art therapy workshops, facilitated by 56 operators. The sample of patients comprised 244 females (62%) and 151 males (38%), aged between 2 and 19 years. The age distribution by gender is illustrated in Table 1.

All patients included in this study were undergoing treatment in a hematology–oncology unit. A specific diagnostic label was available for 119 of them, while the specific oncologic diagnosis was unknown for the remaining 276, in order to satisfy parents’ requests to maintain their children’s diagnosis generic. The most common diagnoses were acute lymphoblastic leukemia—ALL (18 cases), brain tumor (14 cases), acute lymphoblastic leukemia (10 cases), medulloblastoma (9 cases), lymphoma (9 cases), and Wilms tumor (9 cases).

Some art therapy workshops included individuals with diagnoses unrelated to oncological conditions; although these cases were initially considered during data collection, they were excluded from the final sample.

Many patients took part in a full therapeutic path, including several sessions of art therapy. However, for each participant, only the first session was considered.

Caregivers were informed about the observational study, and informed consent was provided and explained prior to enrollment. Patients and caregivers were included in the study only after the consent form was signed by parents or legal guardians.

The art therapy sessions varied in structure and artistic media, encompassing drawing, painting, sculpting, and other creative expressions. These workshops aimed to engage patients in tactile and visual activities that foster emotional expression and therapeutic relaxation. Several hospitals and care facilities across Italy participated in the data collection (Table 2). The art therapy activities were observed live in two facilities, while the remaining sessions were observed online via live video call.

## 3. Results

### 3.1. Score Distribution

**Figure 1:**
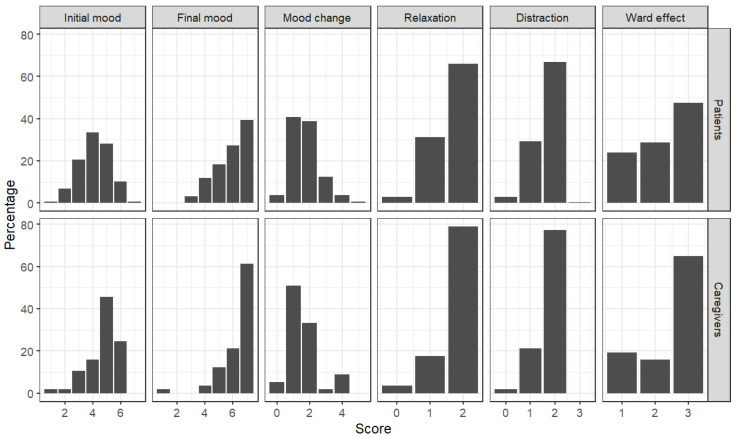
Bar plots illustrating the distribution of scores for each measure. The vertical axis indicates the percentage of participants corresponding to each observed value.

The distributions of the scores (Figure 1) were analyzed to examine the evaluators’ use of the response scale. Specific attention was given to identifying potential asymmetries or biases in scoring patterns. This analysis aimed to provide insights about how well the scale captured variations in mood, relaxation, distraction, and ward effect among the participants.

With regard to the mood scores, the patients’ distribution at the beginning was approximately symmetric, centered on scores of four and five; differently, the distribution of the caregiver scores was most asymmetric, with a peak at scores of five and six. After the intervention (final mood), both distributions became strongly asymmetric, shifting toward positive values. A clear peak at a score of seven was observed, indicating a general improvement in mood after the interventions. The mood change was more pronounced for patients, whose scores increased from an initial range of 4–5 to a maximum score of 7.

Both the relaxation and distraction measures showed a relevant proportion of patients positioned at the central point of the scale. Caregivers, however, were more likely to score at the higher end of the scale. While many patients also reached the highest scale points, this was observed in a proportionally smaller number compared to caregivers.

### 3.2. Inter-Rater Agreement

Analyses were conducted on 38 observations jointly assessed by the EG–MGV pair of observers (30 patients and eight caregivers) and 8 observations jointly assessed by the EG–DDR pair (7 patients and one caregiver).

The inter-rater agreement was evaluated by using two indices.

Matching agreement: This index defines the agreement as an exact match between scores, treating any discrepancy as disagreement. This strict criterion was chosen because agreement requires not just correlation but precise score alignment. However, due to its simplicity, this method has a side effect: measures with a wider scoring range are more susceptible to mismatches. As a result, lower agreement percentages are more likely for mood scores, which use a seven-point scale. The matching agreement was calculated considering only the pair EG-MGV.

Intraclass Correlation Coefficient (ICC), type 1: ICC(1) values, ranging from 0 to 1, provide an additional reliability index, where higher values indicate greater agreement. ICC(1) estimates the proportion of total variance in observed scores that is attributable to differences between subjects, as opposed to variance due to measurement error or inconsistency among raters.

Table 3 presents the results of the analysis. The inter-rater agreement appears generally high, except that for mood change, which—being derived from two separate scores—may reflect error from both sources (in any case, the discrepancies between evaluators are always within one point on the response scale). Many of the ICC(1) values are above 0.6, which is generally sufficient to consider the measures reliable.

### 3.3. Convergent Validity

To evaluate convergent validity, the FACES Pain Rating Scale [18] was administered to a subsample of patients (*n* = 95) and caregivers (*n* = 33). We performed a correlation analysis between the ArtsObs measures and the FACES scores. The results are reported in Table 4.

The correlation coefficients ranged between −0.43 and −0.61 for the patients, indicating moderately strong associations. This result supports the convergent validity of the measure. We observed lower values for caregivers, with indices ranging between −0.22 and −0.41.

### 3.4. Internal Consistency

A correlation analysis using Spearman’s index—suitable for ordinal variables—was conducted between the measures of the ArtsObS for both patient and caregiver data (Table 5).

The relationship between initial mood and final mood is negative. This occurs because, when the initial mood is very low, large increases can be observed in the variable at the end of the intervention; conversely, when the initial mood is already high, the increase observed at the end is not as substantial, even due to a ceiling effect on the response scale. Except for the mood change described above, the scores show adequate levels of correlation.

### 3.5. Sensitivity to Change

The tool’s ability to capture any mood changes resulting from the interventions was studied. Data from both patients and caregivers were combined. The two populations, patients and caregivers, were analyzed separately.

**Figure 2:**
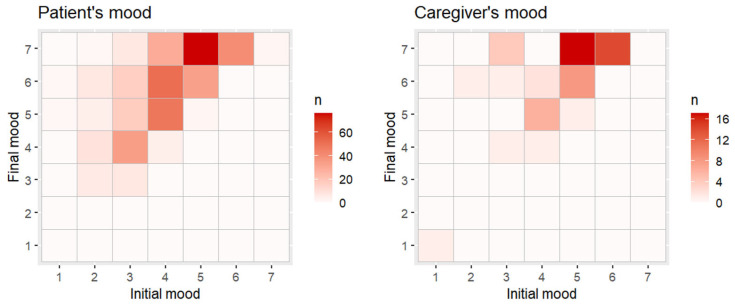
Mosaic plots showing the relation between mood scores (initial mood at the beginning and final mood at the end) for patients and caregivers.

In Figure 2, mood scores at the beginning (horizontal axis) and at the end (vertical axis) of each intervention are shown. For each intersection of scores, a tile is placed, with the color gradient depending on the number of patients with those two values. A linear regression model was applied to verify whether there was a relationship between the mood at the beginning of the activity (independent variable) and the mood at the end of the intervention (dependent variable).

With regard to patients, the model showed a good fit (R^2^ = 0.467), with a significant effect of mood at the start of the intervention (*t*_393_ = 18.65, *p* < 0.001). For every 1-point increase in initial mood, the final mood increased by 0.72 points. The addition of a quadratic term did not lead to a significant improvement, with a null change in R^2^ and a non-significant quadratic coefficient (*t*_392_ = 0.21, *p* = 0.833).

The results were similar for caregivers. The model showed an adequate fit, although lower than that for patients (R^2^ = 0.387), with a significant effect of mood at the start of the intervention (*t*_55_ = 5.89, *p* < 0.001). For every 1-point increase in initial mood, the final mood increased by 0.62 points. The addition of a quadratic term did not lead to a significant improvement, with a small change in R^2^ (R^2^ Difference = 0.027) and a non-significant quadratic coefficient (*t*_54_ = −1.58, *p* = 0.121).

## 4. Discussion

Children often face significant challenges in expressing their emotions verbally [2]. Art, as one of the most ancient and universal forms of communication, offers a valuable outlet for the expression of emotional states and inner conflicts. It thus serves as an effective therapeutic tool in alleviating psychological distress in both pediatric and adult populations [19,20,21]. In healthcare settings, assessing the emotional impact of art-based interventions is critical to substantiating the role of creative modalities in integrated care models [12].

A wide range of methods has been used to evaluate the effectiveness of art therapy interventions, including self-developed questionnaires, Visual Analogue Scales (VAS) [18], semi-structured interviews, and qualitative behavioral observations. However, these tools present several limitations. Ad hoc questionnaires often lack psychometric validation, may introduce bias, and rarely allow comparability across studies. Moreover, both validated and unvalidated self-report tools can be burdensome for patients, particularly children. Qualitative interviews, while rich in content, are time-consuming, require specialized personnel, and often lack the consistency necessary for longitudinal tracking.

The Arts Observational Scale (ArtsObS) was developed to address these methodological gaps. In its Italian adaptation—reported here for the first time to our knowledge, and the first translation of the instrument to another language—the scale enables the noninvasive and efficient monitoring of emotional responses to creative interventions, allowing the care team to monitor psychosocial well-being over the course of treatment, particularly in populations for whom traditional self-report instruments are not feasible. It is quick to administer, requires minimal training, and is suitable for fast-paced or resource-constrained clinical environments. Furthermore, it allows for the simultaneous collection of qualitative and quantitative data that can be benchmarked against institutional goals and used for quality improvement initiatives.

This validation study was conducted using a multicenter observational design and involved 512 individuals, including pediatric patients, caregivers, and facilitators. The Italian version of the ArtsObS was adapted and validated using procedures consistent with the original validation protocol [13], showing comparable psychometric characteristics: high internal consistency, adequate inter-rater reliability, balanced score distributions before and after the intervention, and good concurrent validity with an external criterion (the FACES Pain Rating Scale) [18].

The sample included a diverse range of clinical conditions, ages, and genders, reflecting the real-world variability of the target population. No statistically significant differences emerged in relation to demographic variables, suggesting that the tool performs consistently across a broad spectrum of demographic and clinical profiles.

A methodological limitation could have been the lack of blinding among the evaluators. However, this is consistent with the design of the original study and reflects the inherently observational nature of the tool; the evaluator must observe the participant during or immediately after the art activity. In this context, blinding is neither feasible nor appropriate.

Slight asymmetries in score distributions—particularly in caregiver evaluations, which tended to be more favorable at baseline—were observed. Nevertheless, regression analyses showed measurable improvements across all domains (mood, distraction, relaxation) following the intervention. Although the effect sizes were smaller for caregivers than for patients, the direction and consistency of the changes provide further support for the sensitivity of the tool.

The inter-rater agreement, while moderate in statistical terms, showed minimal score deviation, with most discrepancies limited to a single point on the scale. This low magnitude of disagreement reinforces the scale’s reliability in routine clinical use. Furthermore, the observed correlations between ArtsObS domains and the Pain Rating Scale demonstrate strong convergent validity; both tools capture different but complementary aspects of the emotional and psychological states of pediatric patients.

In addition to numeric scores, the ArtsObS allows for the integration of verbal and nonverbal feedback from patients, caregivers, and healthcare providers. Evaluators are encouraged to record spontaneous or elicited comments, further enriching the dataset with valuable contextual information while maintaining a non-intrusive and patient-centered approach.

The high degree of similarity between the Italian and English versions of the scale suggests that the ArtsObS may be relatively unaffected by cultural or linguistic differences. This opens the door to future cross-cultural studies and the development of international, multicenter research projects aimed at evaluating the psychosocial impact of art therapy in pediatric oncology.

Future research could explore the longitudinal impact of emotional changes—captured through the ArtsObS—on broader clinical outcomes, such as quality of life (QoL) in both children and their caregivers. Establishing such correlations could help reinforce the integration of creative arts therapies into standard psychosocial care protocols for pediatric patients with complex medical needs.

## 5. Conclusions

This research aligns with the growing recognition of creative interventions in improving the quality of life in healthcare contexts. Through adapting the ArtsObS for Italian-speaking populations, this study aimed to fill a critical gap regarding the tools available for evaluating art therapy outcomes in Italy.

The Italian version of the ArtsObS demonstrated good reliability, sensitivity to change, and concurrent validity with traditional pain scores.

It effectively captured variations in mood, relaxation, and distraction, highlighting its value in supporting psycho-emotional well-being during pediatric hospitalization. Its unobtrusive nature and ease of use make it suitable for clinical environments, even with limited resources.

Furthermore, these results could contribute to international efforts to standardize and validate creative methodologies within healthcare systems, ultimately promoting the integration of art therapy as a valuable component of patient care.

## Figures and Tables

**Table 1:** Sample sizes of patients by age and sex.

Age (Years)	Total	Females	Males
1–5	46	26	20
6–9	129	78	51
11–13	77	40	37
14–17	102	83	19
18–19	7	3	4
Missing	34	14	20

**Table 2:** Hospital and care facilities where art therapy sessions were observed.

Facility	Type of Observation	Area	*n* Patients
WonderLAD—NGO	Live	South	67
University-Hospital Polyclinic, Catania	Live	South	38
“La Vela” NGO Social Cooperative	Online	South	3
Vito Fazzi Hospital—Lecce	Online	South	5
Bambino Gesù Children Hospital	Online	Center	39
Pescara Hospital	Online	Center	7
Perugia Hospital	Online	Center	19
University-Hospital Polyclinic—Modena	Online	North	15
Pietro Barilla Children Hospital—Parma	Online	North	5
Pediatric Oncohematology Clinic—Padova	Online	North	5
Filippo del Ponte Hospital—Varese	Online	North	134
“Ponte del Sorriso” NGO Social Cooperative	Online	North	8
Santa Chiara Hospital—Trento	Online	North	28
Home	Online		22

**Table 3:** Agreement between observers.

Measure	Matching Agreement	ICC (1)
Initial mood	0.71	0.78
Final mood	0.76	0.85
Mood change	0.66	0.42
Relaxation	0.92	0.55
Distraction	0.95	0.81
Ward effect	1.00	0.76

**Table 4:** Spearman’s correlation indices between ArtsObs measures and FACES scores.

Measure	Patients	Caregivers
Initial mood	−0.46	−0.42
Final mood	−0.47	−0.26
Relaxation	−0.61	−0.33
Distraction	−0.59	−0.22
Ward effect	−0.43	−0.41

**Table 5:** Spearman’s correlation indices between ArtsObs measures. Values under the main diagonal refer to patients, while values above the diagonal refer to caregivers. Pairwise deletion was used to manage missing data.

	Initial Mood	Final Mood	Mood Change	Relaxation	Distraction	Ward Effect
Initial mood		0.58	−0.36	0.30	0.27	0.12
Final mood	0.71		0.48	0.55	0.64	0.38
Mood change	−0.26	0.43		0.46	0.54	0.29
Relaxation	0.40	0.70	0.51		0.76	0.44
Distraction	0.38	0.70	0.55	0.83		0.58
Ward effect	0.34	0.67	0.50	0.67	0.70	

## Data Availability

The data presented in this study are available on request from the corresponding author. The data are not publicly available due to privacy and ethical restrictions.
